# Transcriptome and Proteome Analysis Revealed That Hormone and Reactive Oxygen Species Synergetically Regulate Dormancy of Introgression Line in Rice (*Oryza sativa* L.)

**DOI:** 10.3390/ijms24076088

**Published:** 2023-03-23

**Authors:** Naihui Guo, Shengjia Tang, Jiayu Wang, Shikai Hu, Shaoqing Tang, Xiangjin Wei, Gaoneng Shao, Guiai Jiao, Zhonghua Sheng, Peisong Hu

**Affiliations:** 1 Rice Research Institute, Shenyang Agricultural University, Shenyang 110866, China; 2 State Key Laboratory of Rice Biology, Key Laboratory of Rice Biology and Breeding, Ministry of Agriculture, China National Rice improvement Centre, China National Rice Research Institute, Hangzhou 310006, China

**Keywords:** dormancy, transcriptome, proteomics, hormone, reactive oxygen species, rice

## Abstract

Dormancy is a complex agronomy phenotype controlled by multiple signaling and a key trait repressing pre-harvest sprouting (PHS). However, the signaling network of dormancy remains unclear. In this study, we used Zhonghua11 (ZH11) with a weak dormancy, and Introgression line (IL) with a strong dormancy to study the mechanism of hormones and reactive oxygen species (ROS) crosstalk regulating rice dormancy. The germination experiment showed that the germination rate of ZH11 was 76.86%, while that of IL was only 1.25%. Transcriptome analysis showed that there were 1658 differentially expressed genes (DEGs) between IL and ZH11, of which 577 were up-regulated and 1081 were down-regulated. Additionally, DEGs were mainly enriched in oxidoreductase activity, cell periphery, and plant hormone signal transduction pathways. Tandem mass tags (TMT) quantitative proteomics analysis showed 275 differentially expressed proteins (DEPs) between IL and ZH11, of which 176 proteins were up-regulated, 99 were down-regulated, and the DEPs were mainly enriched in the metabolic process and oxidation-reduction process. The comprehensive transcriptome and proteome analysis showed that their correlation was very low, and only 56 genes were co-expressed. Hormone content detection showed that IL had significantly lower abscisic acid (ABA) contents than the ZH11 while having significantly higher jasmonic acid (JA) contents than the ZH11. ROS content measurement showed that the hydrogen peroxide (H_2_O_2_) content of IL was significantly lower than the ZH11, while the production rate of superoxide anion (O_2_^.−^) was significantly higher than the ZH11. These results indicate that hormones and ROS crosstalk to regulate rice dormancy. In particular, this study has deepened our mechanism of ROS and JA crosstalk regulating rice dormancy and is conducive to our precise inhibition of PHS.

## 1. Introduction

Transcriptome analysis plays a vital role in deciphering the structure and function of the genome [1]. Due to the continuous reduction of its cost, transcriptome analysis is the preferred way to study the growth, development, and response to the stress of many plants [2]. For example, the ethylene response factor *TaERF87*, a new gene for drought resistance in wheat [3], and the polyamine oxidase *OsPAO5*, a new gene for rice mesocotyl elongation [4], were identified by RNA-seq technology. However, compared with RNA, protein is the direct controller of biochemical processes in organisms, so added protein quantitative techniques, mainly isobaric tags for relative and absolute quantification (iTRAQ) [5,6] and (tandem mass tag) TMT [7,8] techniques, have been applied in plant research. In addition, combining transcriptome and proteome analysis is the best method to analyze gene expression comprehensively and deeply [9]. Transcriptome and proteome analyses revealed that DNA and histone methylation regulated strawberry and tomato fruit ripening [10]. Likewise, transcriptome and proteome analysis revealed the molecular regulatory network of rice leaf vascular development [2]. However, there is little research on the molecular network of rice seed dormancy regulation using the integration of transcriptome and proteome.

Seed dormancy is an important agronomic trait of crops. Its regulation mechanism is complex. Additionally, seed dormancy is an agronomic trait that lacks a thorough understanding [11]. Notably, its complex regulatory mechanism reflects the complexity of seed dormancy. Studies have shown that seed dormancy is regulated by various signals [12,13], including reactive oxygen species (ROS) [14]. ROS is known as a class of oxygen-bearing and highly reactive molecules, including H_2_O_2_, hydroxyl radical (OH), O_2_^.−^, and singlet oxygen (^1^O_2_) [15]. A high concentration of ROS will oxidize cells and cause damage, which is not conducive to germination. In contrast, a low concentration of ROS cannot activate the signal cascade related to germination [14,16,17]. Therefore, maintaining the ROS balance in seeds is critical to promoting germination. Moreover, ROS selectively oxidizes proteins and mRNAs to act as signaling factors to positively regulate seed germination [18]. For example, heat shock proteins and elongation factors in Arabidopsis or peas [19,20] and twenty mRNAs in sunflowers are recognized as signals for seed germination after oxidation by ROS [21].

In addition to directly interacting with the cell wall polysaccharides to promote the elongation of seed radicles [22], ROS can also interact with plant hormones to regulate seed dormancy [23]. Notably, abscisic acid (ABA) inhibits seed germination [24], while gibberellins (GA) antagonize ABA to promote germination [11,25,26]. Additionally, the rice salt tolerance gene *qSE3* positively regulates seed germination under salt stress by increasing ABA content and decreasing H_2_O_2_ content [27]. In barley, the balance between ABA and ROS plays an important role during seed germination [28]. On the one hand, ROS can participate in seed dormancy and germination by participating in ABA and GA metabolism, such as H_2_O_2_ degrading ABA [29,30] and ROS promoting GA synthesis [31]. On the other hand, ABA and GA can also affect the accumulation of ROS. For example, increasing GA will increase the ROS content in Avena fatua [32], while ABA will reduce the ROS content in sunflowers [33]. Recently, studies have shown that JA also participates in seed germination. ABA promotes jasmonic acid (JA) synthesis, thus releasing abscisic acid insensitive (*ABI5*) to activate the ABA signaling pathway, and finally inhibiting seed germination [34]. In turn, JA will inhibit the synthesis of ABA to promote seed germination [35]. However, it is unknown whether JA and ROS will crosstalk to regulate seed dormancy and germination.

Through transcriptome and proteome analysis, this study found that hormone- and ROS-related gene expressions were significantly different in strongly dormant IL than in weak dormant ZH11. The determination of hormone and ROS content found that compared with ZH11, the content of ABA, JA, and ROS in IL was significantly different. Our work contributes to further understanding the complexity of dormancy responses and provides insights into the multi-signal crosstalk of dormancy in rice and other crops.

## 2. Results

### 2.1. The Germination Rate of IL Was Significantly Lower Than That of ZH11

**Figure 1:**
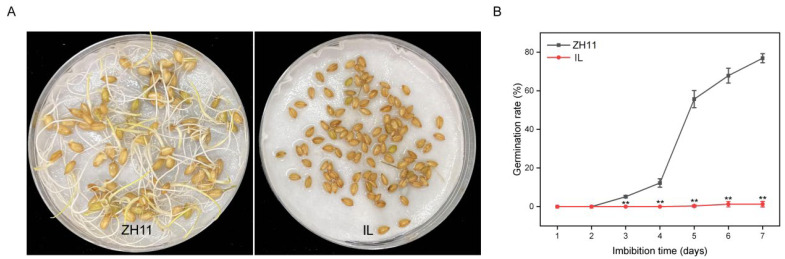
Germination phenotype identification of ZH11 and IL. (**A**) Germination phenotype of ZH11 and IL on the seventh day of germination soon after harvest. (**B**) Germination percentage of ZH11 and IL after harvest. The data represent the mean ± SD (*n* = 3), ** *p* ≤ 0.01.

We conducted germination experiments with seeds 35 DAH. The germination rate of IL on the seventh day was 1.25%, whereas that of ZH11 on the seventh day was 76.86% (Figure 1). Thus, the germination rate of IL was significantly lower than that of ZH11 by 75.61%.

### 2.2. Transcriptome Differences between ZH11 and IL

To understand the regulatory pathway differences between ZH11 and IL dormancy, we detected the transcriptome of seeds during the six hours of imbibition. Six samples were sequenced in this study, with three repeats for ZH11 and IL, respectively. According to the expression level of each sample in FPKM, the violin chart shows the distribution of gene expression level and data distribution of each sample (Supplementary Figure S1A). The principal component analysis (PCA) results showed that the sample was repeatable, and there was a significant difference between ZH11 and IL (Supplementary Figure S1B). The Pearson correlation coefficient reflected the similarity of overall gene expression among each sample (Supplementary Figure S1C).

**Figure 2:**
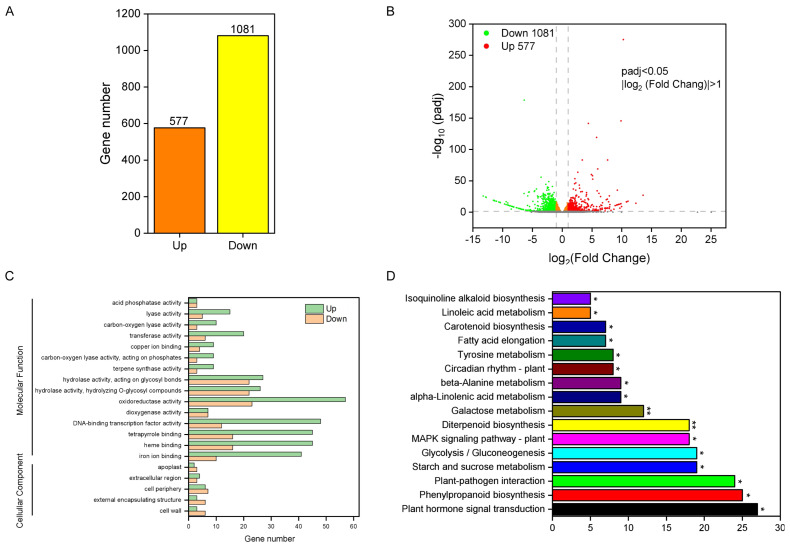
Transcriptome analysis of IL and ZH11. (**A**) The number of up- and down-regulated genes (padj < 0.05) in the seeds following six hours of imbibition of IL compared with ZH11. (**B**) Volcano plot of DEGs between IL and ZH11. (**C**) Go analysis of DEGs (padj < 0.05), divided into cellular composition and molecular function. (**D**) KEGG pathway enrichment analysis of DEGs. The data represent the mean ± SD (*n* = 3), * *p* ≤ 0.05 and ** *p* ≤ 0.01.

A total of 1658 DEGs were detected (padj < 0.05), of which 34.8% (577 genes) were up-regulated and 65.2% (1081 genes) were down-regulated in the IL compared with the expression levels in ZH11 (Figure 2A,B). To verify the reliability of RNA-seq data, 8 genes were selected for qRT-PCR analysis. The results showed that the expression of RNA-seq and qRT-PCR of differential genes were similar (Supplementary Figure S2), which suggests that the results of RNA-seq were reliable. GO analysis reveals DEGs enrichment in molecular function and cellular components. Oxidoreductase activity, heme binding, and tetrapyrrole binding represented 15.21, 11.6, and 11.6% of total DEGs and were the largest subcategories in the molecular function. Cell periphery, cell wall, and external encapsulating structure represented 30.23, 20.93, and 20.93% of total DEGs, respectively, and were the largest subcategories in the cellular component (Figure 2C). In KEGG enrichment, a total of 477 DEGs were enriched in 88 pathways, of which 16 pathways were significantly enriched (Figure 2D). Plant hormone signal transduction, phenylpropanoid biosynthesis, plant-pathogen interaction, and glycolysis/gluconeogenesis were the most significantly enriched pathways of the 16 pathways (Figure 2D). Therefore, the results demonstrated that DEGs enriched in oxidoreductase activity, cell periphery, and plant hormone signal transduction pathways may be the reasons for promoting IL dormancy.

### 2.3. Proteomic Profiles Differences between ZH11 and IL

**Figure 3:**
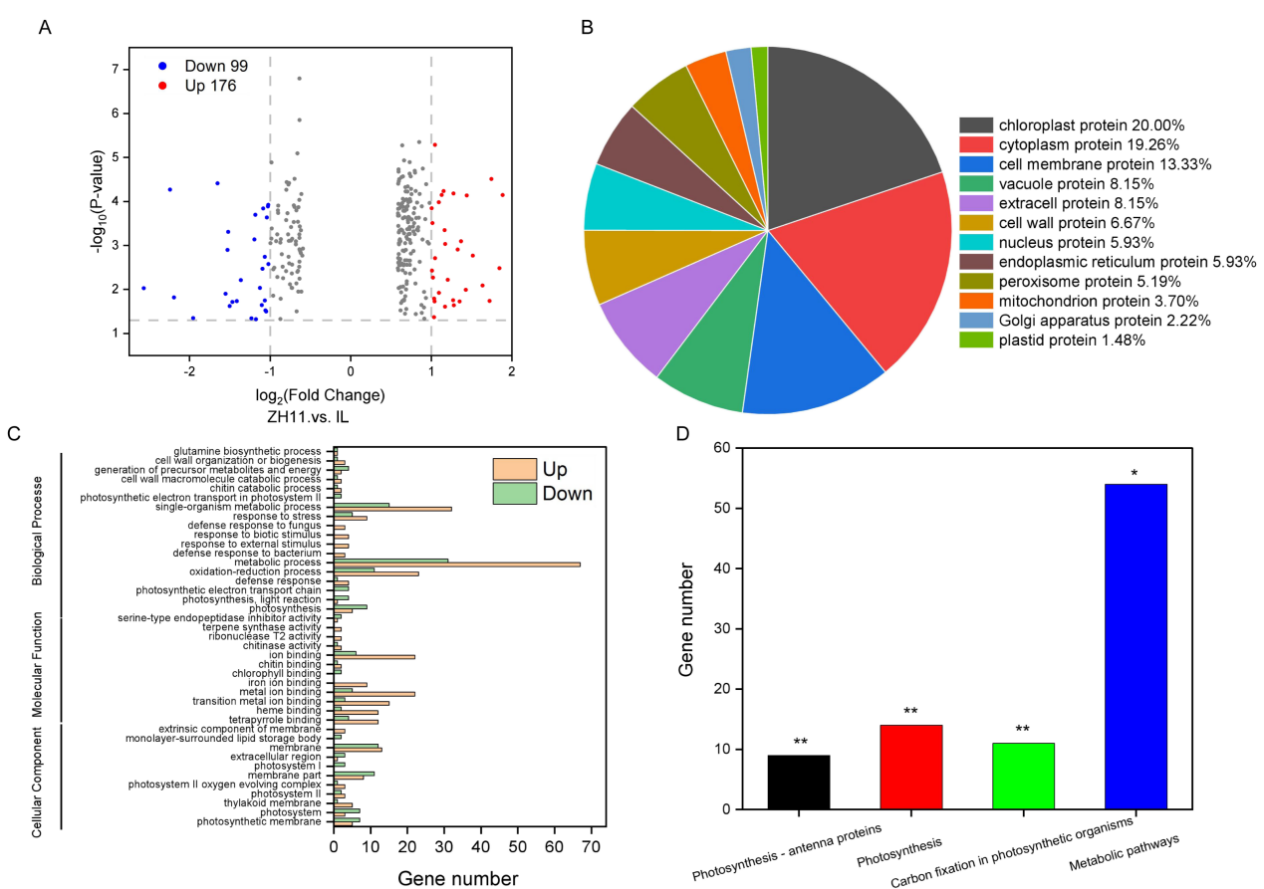
Proteome analysis of IL and ZH11. (**A**) Volcano plot of DEPs between IL and ZH11. (**B**) Subcellular localization analysis of DEPs. (**C**) Go analysis of DEPs (padj < 0.05), divided into cellular composition, molecular function, and biological process. (**D**) KEGG pathway enrichment analysis of DEPs. The data represent the mean ± SD (*n* = 3), * *p* ≤ 0.05 and ** *p* ≤ 0.01.

TMT quantitative proteomics was used to analyze the seeds on the six hours of imbibition between ZH11 and IL to identify the protein involved in dormancy. The results of the PCA showed that the sample was repeatable, and there was a significant difference between ZH11 and IL (Supplementary Figure S3). A total of 275 DEPs were detected (padj < 0.05), of which 64.0% (176 genes) were up-regulated and 37.0% (99 genes) were down-regulated in the IL compared with the expression levels in ZH11 (Figure 3A).

To identify the functions of DEPs, we annotated the proteins to the subcellular localization, GO, and KEGG databases. There 135 DEPs were identified in the subcellular localization database, mainly including chloroplast protein (20.00%), cytoplasm protein (19.26%), and cell membrane protein (13.33%) (Figure 3B). DEPs enrichment was noted in cellular components, molecular functions, and biological processes using GO analysis. The same protein, such as A3BKU8 and Q7G3F1, appears in multiple teguments, indicating that it plays a role in many metabolic pathways. With the 93 DEPs involved in cellular components, the main categories represented were membranes (69.89%) and photosystems (23.66%) (Figure 3C). With the 127 DEGs involved in molecular function, the main categories represented were binding ability (92.13%) and enzyme activity (7.87%) (Figure 3C). Similarly, in the biological processes containing 255 DEPs sub-ontology, 38.43, 18.43, and 13.33% of the DEPs were metabolic processes, single-organism metabolic processes, and oxidation-reduction processes, respectively (Figure 3C). Likewise, there 88 DEPs were identified in the KEGG enrichment, of which 61.36% were involved in metabolic pathways, 26.14% were involved in photosynthesis (26.14%), and 12.50% were involved in carbon fixation in photosynthetic organisms (Figure 3D). The results showed that DEPs enriched in metabolic processes (such as Q84NW1 and Q8LID9) and oxidation-reduction processes (such as B9FUG0 and Q6ZFJ3) may be the reasons for promoting IL dormancy.

### 2.4. Association Analysis of RNA-Seq and Proteomics

**Figure 4:**
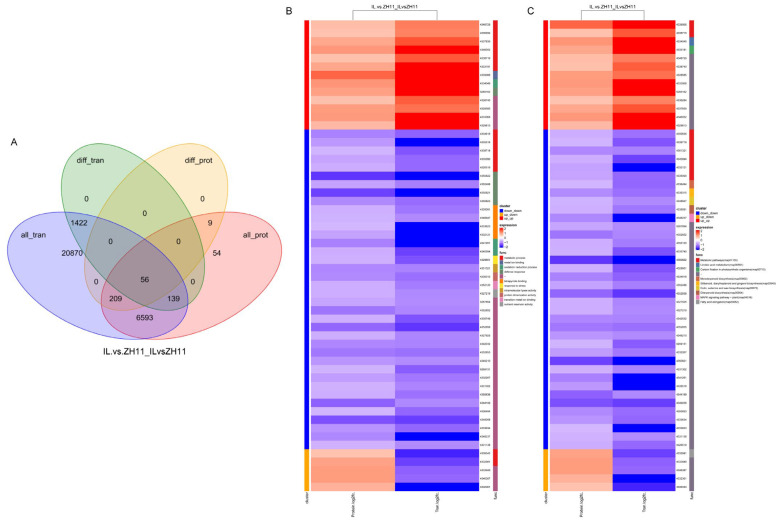
Association analysis of RNA-seq and proteomics. (**A**) Overlap between transcript and protein. (**B**) Go enrichment analysis of the heat map of the RNA-seq and proteomics. (**C**) KEGG enrichment analysis of the heat map of the RNA-seq and proteomics.

To comprehensively analyze the transcriptome and proteome, we performed an association analysis. As a result, 56 differentially expressed genes were identified in both transcriptome and proteome, accounting for 3.46% of DEGs in the transcriptome and 20.44% of DEPs in the proteome (Figure 4A). GO analysis showed that DEGs were mainly enriched in the metabolic, metal ion binding, and oxidation-reduction processes (Figure 4B). KEGG analysis showed that DEGs were primarily enriched in metabolic pathways, linoleic acid metabolism, and carbon fixation in photosynthetic organisms (Figure 4C). Comprehensive analysis showed that transcriptome and proteome were less correlated.

### 2.5. Hormones Affect the Dormancy of IL

**Figure 5:**
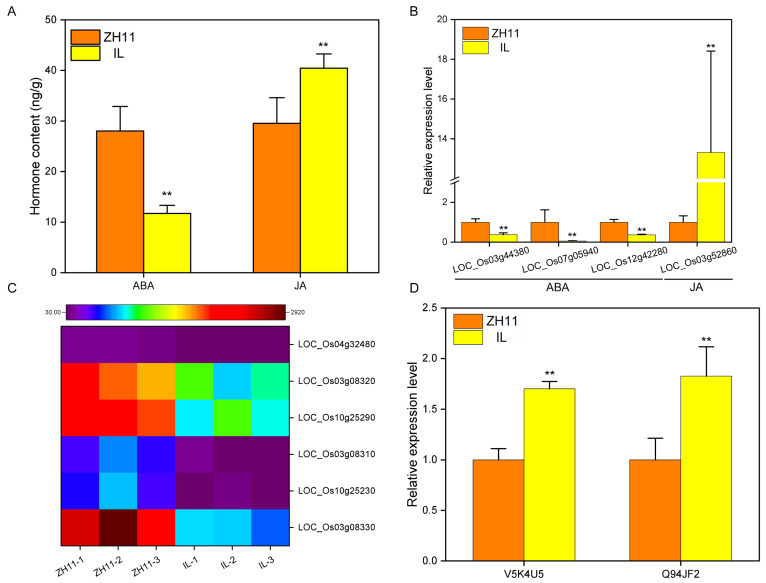
Analysis of hormone content and signal transduction pathways. (**A**) Contents of abscisic acid and jasmonic acid in the seeds of IL and ZH11 following six hours of imbibition. (**B**) Relative expression of abscisic acid and jasmonic acid synthesis genes in the seeds of IL and ZH11 following six hours of imbibition. (**C**) DEGs of signal transduction genes of jasmonic acid in IL and ZH11. (**D**) DEPs of signal transduction proteins of abscisic acid in IL and ZH11. The data represent the mean ± SD (*n* = 3), ** *p* ≤ 0.01.

We have noticed that in the KEGG enrichment of RNA-seq, most DEGs were enriched in plant hormone signal transduction (Figure 2D), and hormones are closely related to dormancy [36]. Thus, we measured the hormone content of ZH11 and IL and analyzed the difference in hormone synthesis and signal transduction gene expression. Compared with ZH11, the content of the ABA in IL decreased by 58.19%, and the JA increased by 36.92% (Figure 5A). Further analysis showed that the expression of key genes *LOC_Os03g44380*, *LOC_Os07g05940*, and *LOC_Os12g42280* of the ABA synthesis pathway in IL was significantly lower than that in ZH11. In contrast, the expression of the first gene *LOC_Os03g52860* of the JA synthesis pathway in IL was considerably higher than that in ZH11 (Figure 5B). In addition, proteome analysis showed that the content of protein P29250 (the protein of *LOC_Os03g52860*) was significantly higher in IL than in ZH11 (Supplementary Figure S4). Simultaneously, the expression levels of JA signal transduction genes *LOC_Os04g32480*, *LOC_Os03g08320*, *LOC_Os10g25290*, *LOC_Os03g08310*, *LOC_Os10g25230*, and *LOC_Os03g08330* and ABA signal transduction proteins V5K4U5 and Q94JF2 were significantly different between ZH11 and IL (Figure 5C,D). These results suggested that ABA and JA were crucial factors affecting the dormancy of IL.

### 2.6. Reactive Oxygen Species Affect the Dormancy of IL

**Figure 6:**
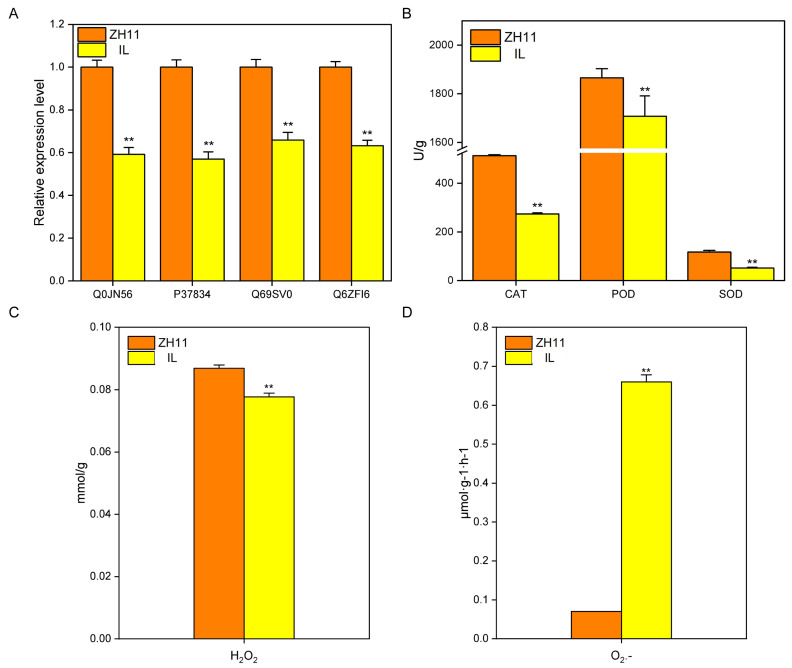
Analysis of reactive oxygen species pathways. (**A**) DEPs of peroxisome proteins in IL and ZH11. (**B**) ROS scavenging enzyme content. “U” for CAT: the degradation of 1 μmol H_2_O_2_ per minute per gram of tissue in the reaction system is defined as an enzyme activity unit; “U” for POD: A470 change of 0.01 per minute per gram tissue in each mL reaction system is defined as an enzyme activity unit; “U” for SOD: the amount of enzyme required to inhibit 50% photochemical reduction of Nitrotetrazolium Blue chloride (NBT) was 1 enzyme activity unit. (**C**) H_2_O_2_ content of IL and ZH11. (**D**) O_2_^−^ generation rate of IL and ZH11. The data represent the mean ± SD (*n* = 3), ** *p* ≤ 0.01.

In the subcellular localization database, differential proteins are enriched on peroxisomes (Figure 3B). Thus, we analyzed the protein expression of the peroxisome gene. The results showed that IL’s expression of four peroxisome proteins, Q0JN56, P37834, Q69SV0, and Q6ZFI6, was significantly lower than that in ZH11 (Figure 6A). Due to the difference in peroxisome protein content, we determined the content of CAT, POD, and SOD. The results showed that compared with ZH11, the contents of CAT, POD, and SOD in IL decreased by 46.49, 8.45, and 56.15%, respectively (Figure 6B). Notably, the difference in peroxidase content will inevitably lead to a difference in ROS content [37,38]. As expected, compared with ZH11, the content of H_2_O_2_ in IL was significantly reduced by 10.55%, but the production rate of O_2_^−^ was significantly increased by 8.39 times (Figure 6C,D). Therefore, the above results suggested that ROS is an essential regulatory factor affecting the dormancy of IL.

## 3. Discussion

Seed dormancy has an irreplaceable effect on plants, especially crop cultivation. Consequently, the loss of dormancy can easily lead to pre-harvest sprouting and affect crop quality and yield, while too strong dormancy results in uneven seed germination, which is not conducive to sowing [39]. Therefore, mastering the regulatory mechanism of dormancy is crucial for precise control of dormancy. Unfortunately, little is known about the molecular mechanisms and physiological processes of dormancy. So far, more than 140 dormancy-related quantitative trait loci genes have been identified in rice, but only a few genes have been cloned, such as *Sdr4* [39], *OsVP1* [40], *SD1* [41], *OsNCED3* [42], and *Rc* [43]. In the present study, we constructed IL, which is identical to ZH11 except for the segment on chromosome 3 between markers RM1 and RM2. Compared with ZH11, the germination rate of IL was significantly decreased (Figure 1), indicating that the replaced fragments in IL contained strongly dormant genes. Consistent with this finding, a dormancy QTL was previously reported to be detected in the same interval in wild rice [44]. The above results indicate that the dormancy-related gene can be detected between markers RM1 and RM2 using different rice germplasm resources, a new rice dormancy gene. Thus, our study provides a basis for further cloning new rice dormancy genes and improving the molecular network of dormancy regulation.

Transcriptome analysis plays an important role in the study of rice molecular mechanisms [45,46]. For example, RNA-Seq sequencing detected a significant down-regulation of *OsSUT1* expression in *dao* mutants and further verified that *OsSUT1* can directly regulate the source-sink allocation of sucrose in plants [47]. Transcriptome data analysis also revealed that *OsNCED3* mainly regulates rice dormancy through hormone and signal transduction [42]. This study found that DEGs were primarily enriched in oxidoreductase activity, cell periphery, and plant hormone signal transduction pathways (Figure 2C,D). Further, we used TMT proteome sequencing technology to analyze and found that DEPs were mainly enriched in the metabolic process and oxidation-reduction process (Figure 3). Some previous studies have also demonstrated that the transcriptome and proteome are less correlated [9,48]. For instance, 21,111 genes and 3531 DEGs were identified in tomatoes under high-temperature stress, but only 2297 proteins and 268 DEPs [49]. In this study, 1658 DEGs were detected (Figure 2A), but only 275 DEPs were detected (Figure 3A), and only 56 differential genes were identified in both RNA and protein (Figure 4A). In addition to being relevant to the prior art, this situation is also related to the alternative splicing of RNA [9]. Nevertheless, the differentially expressed genes/proteins in the transcriptome and proteome were enriched in the oxidation-reduction, indicating that oxidation-reduction is an essential reason for the dormancy of IL.

Hormones are an important factor in regulating the dormancy of the seeds. Hormone content showed that the ABA content of IL was significantly lower than that of ZH11, while the content of JA was substantially higher than that of ZH11 (Figure 5A). Meanwhile, transcriptome analysis showed that compared with ZH11, ABA synthesis genes *LOC_Os03g44380*, *LOC_Os07g05940*, and *LOC_Os12g42280* were significantly decreased in IL, and the expression of JA synthesis gene *LOC_Os03g52860* was significantly increased in IL (Figure 5B). These findings demonstrate that IL regulates the content of hormones by affecting the expression of hormone synthesis genes. Generally speaking, high ABA content promotes seed dormancy [50]. Notably, the strong dormancy of IL is inconsistent with its low ABA content, suggesting that ABA content is not the cause of IL dormancy. However, the proteome showed that the expression levels of two ABA signaling proteins were significantly increased in IL compared with ZH11 (Figure 5D). Thus, this implies that enhancement of ABA signaling causes dormancy of IL. Additionally, the regulation of dormancy by JA is twofold. Study has shown that in rice, ABA promotes the expression of JA synthesis gene *AOC* to increase the content of JA and synergistically inhibit seed germination [51]. Another study has also shown that in wheat, JA inhibits the expression of ABA synthesis genes *TaNCED1* and *TaNCED2* to reduce the content of ABA and promote seed germination [35]. The increase of JA content in IL (Figure 5A) indicated that JA was one of the reasons affecting the dormancy of IL. Furthermore, transcriptome analysis revealed significant differences in the expression of JA signaling genes *LOC_Os04g32480*, *LOC_Os03g08320*, *LOC_Os10g25290*, *LOC_Os03g08310*, *LOC_Os10g25230,* and *LOC_Os03g08330* between ZH11 and IL (Figure 5C), further suggesting that JA regulates IL dormancy.

A specific concentration of ROS can act as a signal molecule to regulate seed dormancy, mainly through interaction with hormones. H_2_O_2_ promotes seed germination, activating GA signaling [51] or promoting ABA degradation [30]. It has also been reported that the accumulation of O_2_^−^ can relieve seed dormancy [52]. In this study, compared with ZH11, the content of H_2_O_2_ in IL decreased significantly (Figure 6C), but this did not increase the content of ABA in IL (Figure 5A), indicating that H_2_O_2_ did not regulate IL dormancy by degrading ABA content. However, excessive O_2_^−^ accumulation in IL did not release IL dormancy (Figure 6D), suggesting that O_2_^−^ exceeded the threshold of signal regulation.

## 4. Materials and Methods

### 4.1. Materials Obtained and Field Experiments

ZH11 is a *japonica* with a high germination rate. A low germination rate of *Aus* rice, Mengjialaxiaoli, was crossed with ZH11 and then backcrossed with ZH11 for four generations. Marker-assisted selection was conducted for each generation in segregating progeny with the SSR markers RM1 and RM2. Finally, 123 pairs of SSR polymorphic markers evenly distributed on 12 chromosomes were used for background screening. The single plant whose genotypes of all markers except RM1 and RM2 were consistent with ZH11 was selected as IL. All materials were planted in the experimental field of the China National Rice Research Institute, with normal paddy field management. The primer sequences of RM1 and RM2 are listed in Supplementary Table S1.

### 4.2. Germination Phenotypic Evaluation

About 100 seeds 35 days after heading (DAH) were placed in a 9 cm petri dish covered with two layers of filter paper, and 10 mL ddH_2_O was added to the plates to provide a total of three biological repeats. We then placed the petri dish in an incubator set to 25 °C, dark, and with a constant humidity of 80%. The germination rate was counted every 24 h for 7 consecutive days. The bud length was half the seed length, or the root length was the full length of the seed as germination.

### 4.3. RNA Extraction and qRT-PCR Analysis

RNA extraction and qRT-PCR methods were conducted as previously published [53]. In brief, the total RNA was extracted using Trizol (Invitrogen, Carlsbad, CA, USA), and the RNA was converted into cDNA using the ReverTra Ace qPCR RT kit (Toyobo, Osaka, Japan). Furthermore, using cDNA as a template, RT-PCR analysis was performed using the Applied Biosystems Quant Studio 3 Real-Time PCR System instrument. The primers used in this assay are listed in Supplementary Table S1.

### 4.4. RNA-Seq Analysis

Seeds of ZH11 and IL were harvested after six hours of imbibition and immediately frozen in liquid nitrogen [54]. Beijing Novo Gene Company completed RNA library preparation and sequencing for RNA-seq. RNA was transformed into double-stranded cDNA and sequenced by the Illumina NovaSeq 6000. Differential expression analysis of two groups was performed using the DESeq2 R package (1.20.0). The resulting *p*-values were adjusted using Benjamini and Hochberg’s approach for controlling the false discovery rate. padj < 0.05 and |log_2_(foldchange)| > 1 were set as the threshold for significantly differential expression. Gene Ontology (GO) enrichment analysis of differentially expressed genes was implemented by the clusterProfiler R package (3.8.1), in which gene length bias was corrected. GO terms with corrected *p*-values less than 0.05 were considered significantly enriched by differential expressed genes. Furthermore, the Cluster Profiler R package (3.8.1) was used to test the statistical enrichment of differential expression genes in KEGG pathways.

### 4.5. Tandem Mass Tag (TMT) Quantitative Proteomics

Beijing Novo Gene Company completed proteome determination. This was performed by immersing the sample with SDT (4% Sodium dodecyl sulfate, 10 mM Dithi-othreitol, 100 mM Triethylammonium bicarbonate buffer) protein lysate, and then extracting the protein through ultrasonic wave breaking. Each sample was repeated three times. ZH11-1, ZH11-2, ZH11-3, IL-1, IL-2, and IL-3 are labeled with TMT tags of 127 N, 128 N, 131 N, 132 N, 133 N, and 134 N, respectively. Following labeling with TMT, the fractions were separated using the L-3000 HPLC system. The chromatographic column is Waters BEH C18 (4.6 × 250 mm, 5 μm). The separated peptides were analyzed by Q ExactiveTM series mass spectrometer (Thermo Fisher, Waltham, MA, USA), with an ion source of Nanospray Flex™ (ESI), spray voltage of 2.3 kV, and ion transport capillary temperature of 320 °C. A full scan ranging from *m*/*z* 350 to 1500 with a resolution of 60,000 (at *m*/*z* 200), an automatic gain control (AGC) target value of 3 × 10^6^, and a maximum ion injection time of 20 ms. The top 40 precursors of the highest abundance in the full scan were selected and fragmented by higher energy collisional dissociation (HCD) and analyzed using MS/MS, where the resolution was 45,000 (at *m*/*z* 200) for 10 plex, the automatic gain control (AGC) target value was 5 × 104, the maximum ion injection time was 86 ms, a normalized collision energy was set as 32%, an intensity threshold was 1.2 × 105, and the dynamic exclusion parameter was 20 s. The raw data of MS detection was named “.raw”.

### 4.6. Measurement of Free ABA and JA Content

Seeds of ZH11 and IL following six hours of imbibition were harvested and immediately frozen in liquid nitrogen. The samples were analyzed using the LC-MS/MS system following treatment. An ultra-high performance liquid chromatography coupled to tandem mass spectrometry (UHPLC-MS/MS) system (ExionLCTM AD UHPLC-QTRAP 6500+, AB SCIEX Corp., Boston, MA, USA) was used to quantitate phytohormone in Novogene Co., Ltd. (Beijing, China). Separation was performed on a Waters XSelect HSS T3 column (2.1 × 150 mm, 2.5 μm) maintained at 45 °C. The mobile phase, consisting of 0.01% formic acid in water (solvent A) and 0.01% formic acid in acetonitrile (solvent B), was delivered at a flow rate of 0.30 mL/min. The solvent gradient was set as follows: initial 10% B, 1 min; 10–50% B, 3 min; 50–65% B, 4 min; 65–70% B, 6 min; 70–100% B, 7 min; 100–10% B, 9.1 min; 10% B, 12 min.

The mass spectrometer was operated in multiple reaction mode (MRM) mode. Parameters were set as follows: IonSpray Voltage (Negative mode: −4500 V, Positive mode: 4500 V), Curtain Gas (35 psi), Ion Source Temp (550 °C), Ion Source Gas of 1 and 2 (60 psi).

### 4.7. SOD Activity Assay

The determination method of superoxide dismutase (SOD) content was slightly modified with reference to previous research [37]. We collected 0.1 g samples, added 1 mL of 50 mmol/L phosphate buffer (pH 7.8) pre-cooled at 4 °C, grind to homogenate and centrifuged at 10,000× *g* for 10 min at 4 °C, and the supernatant had a maximum absorption peak at 560 nm. The amount of enzyme required to inhibit NBT photoreduction by 50% was 1 unit of enzyme activity.

### 4.8. POD Activity Assay

The determination method of peroxidase (POD) content was slightly modified with reference to previously published methods [38]. We collected 0.1 g samples, added 1 mL of 50 mmol/L phosphate buffer (pH 7.8) pre-cooled at 4 °C, and ground them evenly. Subsequently, this was centrifuged at 10,000× *g* for 10 min at 4 °C. We then selected the supernatant and measured the OD value at a wavelength of 470 nm. One unit of enzyme activity was defined as a 0.01 change in A470 per minute per gram of tissue per mL of the reaction system.

### 4.9. CAT Activity Assay

The determination method of catalase (CAT) content was slightly modified with reference to previously published methods [37]. We collected 0.1 g samples, added 1 mL of 50 mmol/L phosphate buffer (pH 7.8) pre-cooled at 4 °C, and ground them evenly. We then centrifuged these samples at 10,000× *g* for 10 min at 4 °C, and selected the supernatant to measure the OD value at a wavelength of 240 nm. One unit of enzyme activity was defined as the degradation of 1 μmol H_2_O_2_ per minute per gram of tissue in the reaction system.

### 4.10. O_2_^−^ Production Rate Assay

The determination method of O_2_^−^ production rate was slightly modified with regards to previous research [38]. We collected 0.1 g samples, added 1.5 times the 50 mmol/L phosphate buffer volume to grind and homogenize, and centrifuged these at 10,000× *g*/min for 20 min. The supernatant had a maximum absorption peak of 530 nm.

### 4.11. H_2_O_2_ Content Determination

The determination method of H_2_O_2_ content was slightly modified with reference to previous research [38]. We collected 0.1 g samples, added 1 mL of 50 mmol/L phosphate buffer, mixed thoroughly, and leached for 10 min. Subsequently, we centrifuged the samples at 10,000× *g*/min for 5 min. We then selected the supernatant to measure the OD value at a wavelength of 405 nm.

### 4.12. Statistical Analysis

One-way ANOVA was used to test the statistically significant differences among tested varieties, which was performed using SPSS 22.0 software (IBM Inc., Armonk, NY, USA).

## 5. Conclusions

In summary, the dormancy of IL is jointly regulated by ABA, JA, and ROS signals. Due to the lack of research on ROS cooperating with JA to regulate dormancy, our research undoubtedly fills this gap. However, the signaling network regulating dormancy is delicate and complex, and how ABA, JA, and ROS cooperate to regulate seed dormancy needs further study.

## Data Availability

The raw sequence data (RNA-Seq raw data) have been deposited to the NCBI (https://www.ncbi.nlm.nih.gov/sra/PRJNA942433; accessed on 9 March 2023) with accession numbers PRJNA942433. The mass spectrometry proteomics data have been deposited to the ProteomeXchange Consortium (http://proteomecentral.proteomexchange.org; accessed on 24 February 2023) via the iProX partner repository with the dataset identifier PXD040365.

## Supplementary Materials

The following supporting information can be downloaded at: https://www.mdpi.com/article/10.3390/ijms24076088/s1.
