# Supplementary material for: Transcriptome and Proteome Analysis Revealed That Hormone and Reactive Oxygen Species Synergetically Regulate Dormancy of Introgression Line in Rice (Oryza sativa L.)
Source: Int J Mol Sci. 2023 Mar 23;24(7):6088. doi: 10.3390/ijms24076088 (PMC10094489; doi:10.3390/ijms24076088)
Supplement: Supplementary file 1 [file ijms-24-06088-s001.zip › ijms-2254207-supplementary.pdf]

Supplementary Material

Supplementary Figures

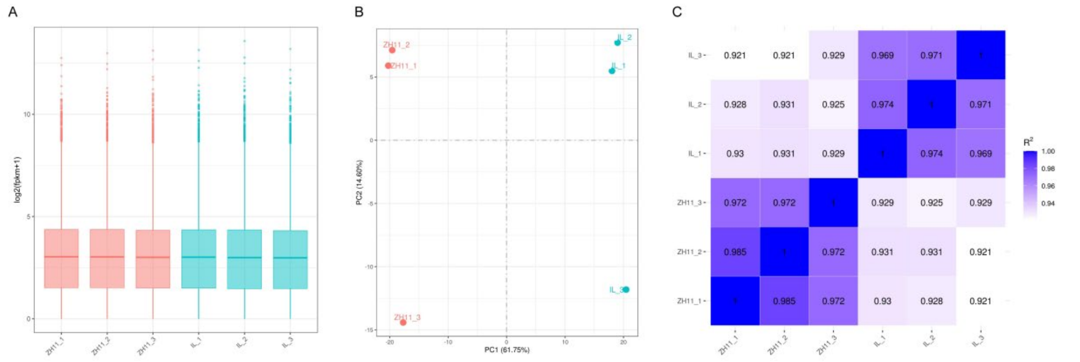

Figure S1. (**A**)Violin plot representation of the gene expression range. (**B**) PCA results of different transcriptome samples. (**C**) Pearson correlation among samples analysis.

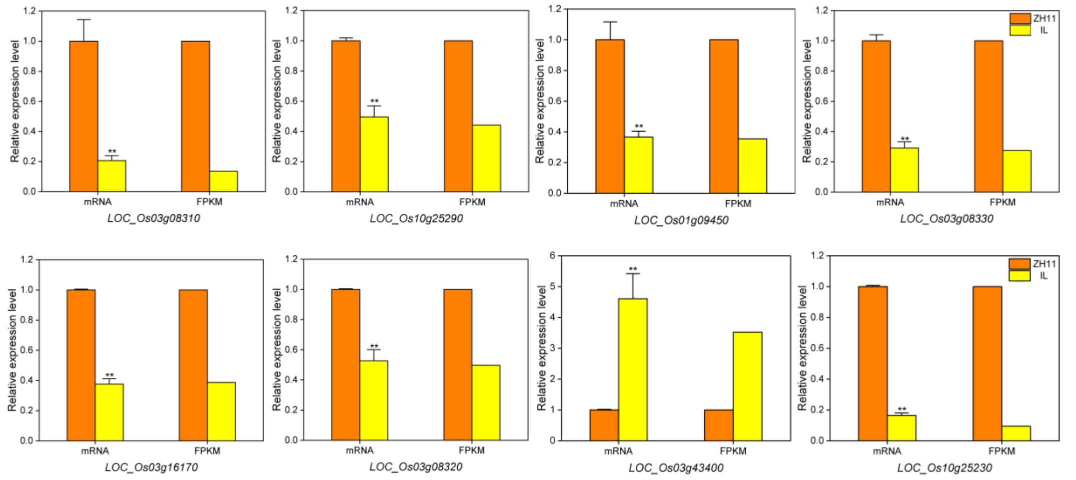

Figure S2. Eight randomly selected genes for qRT-PCR comparison with transcriptome data. The data represented the mean values ± SD (n = 3), **p* ≤ 0.05.

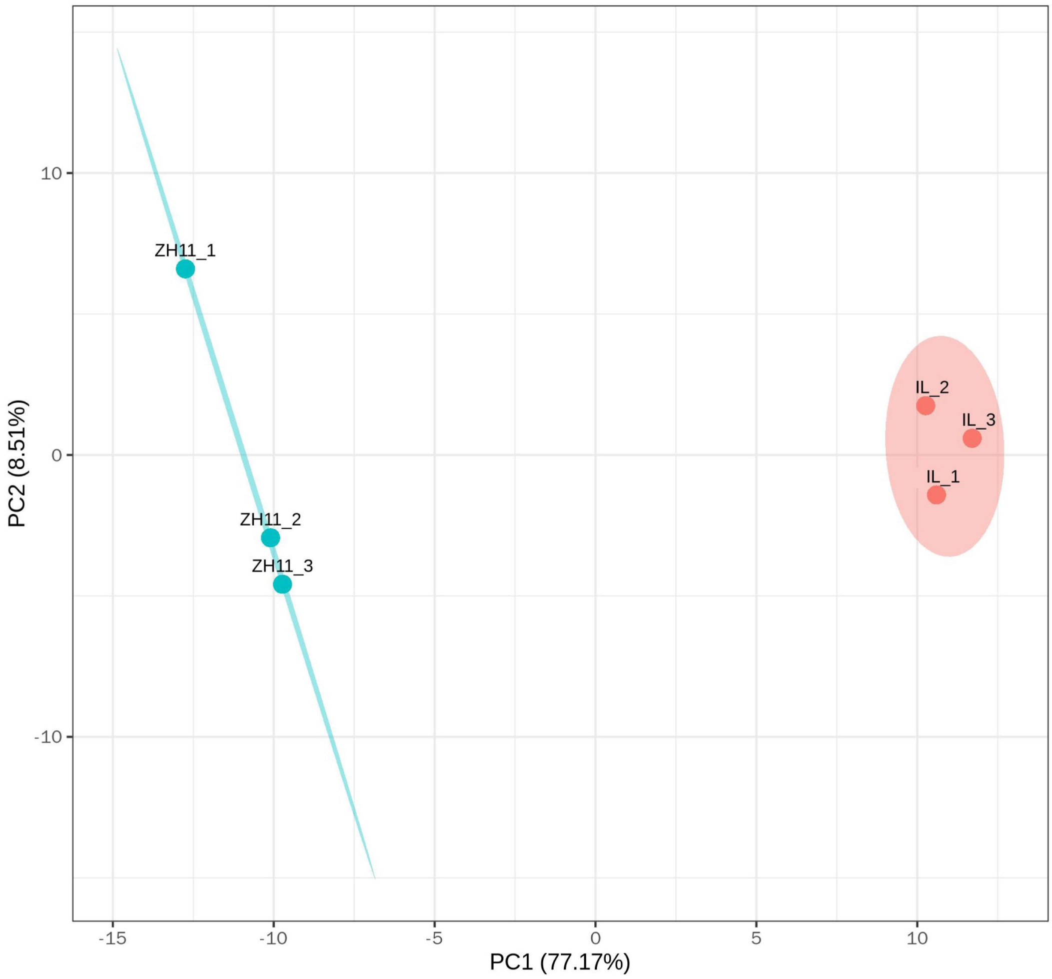

Figure S3. PCA results of different proteome samples.

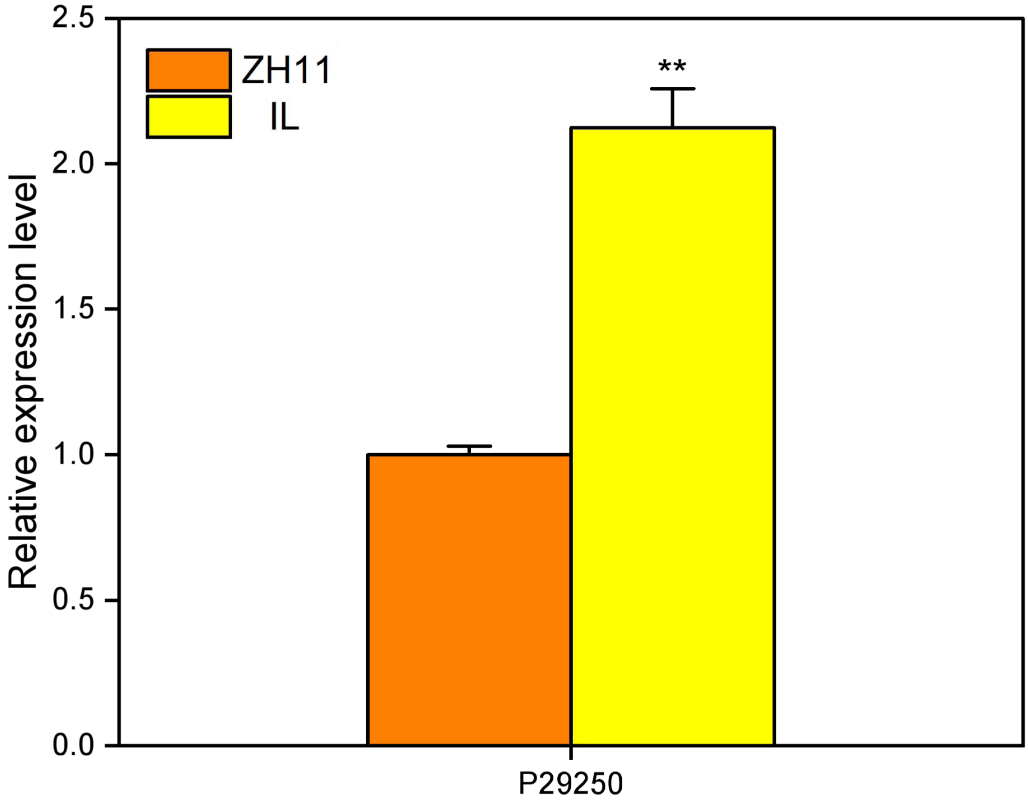

Figure S4. Expression of JA synthesis pathway proteins in IL and ZH11.

Supplementary Table

Table S1. Primers used in this study.

Primers	Sequence (5′-3′)	Description
Actin-F	GTCCTCTTCCAGCCTTCCTT	RT
Actin-R	CTCATCCTGTCAGCAATGCC	
RT-LOC_Os03g08330-F	GATGGAGGGGAAGAGCAGG	
RT-LOC_Os03g08330-R	CCATCCTCCCACCGTAGAAG	
RT-LOC_Os10g25230-F	ATGACCATCTTCTACGGCGG	
RT-LOC_Os10g25230-R	TGCCCTTCCTCTTCTCCATG	
RT-LOC_Os03g08310-F	CAGTACGTCAAGGCCAACTC	
RT-LOC_Os03g08310-R	CGGCGTAGAAGATCGTGAAC	
RT-LOC_Os03g43400-F	ACCTGAGGAAGATCGACGTC	
RT-LOC_Os03g43400-R	CCTTTCATGATTCGGAGGCG	
RT-LOC_Os10g25290-F	TGACCATCTTCTACGACGGG	
RT-LOC_Os10g25290-R	CCGCCTTCTTGTATGGTTCG	
RT-LOC_Os01g09450-F	CTTCTCCTCCTCCTCCTCCT	
RT-LOC_Os01g09450-R	CTGCTTGGACGACGATGAC	
RT-LOC_Os03g16170-F	CCCCTCTCATGCGACCATAA	
RT-LOC_Os03g16170-R	CTCTCCATCACCCTCACCTC	
RT-LOC_Os03g08320-F	ACATGAAGGAGCACAGTGGA	
RT-LOC_Os03g08320-R	TTCTTGCGTGTCTTTCAGCG	
RM1-F	CTGCCCTGCCTTTTGTACAC	IL

RM1-R	GCGAGCATTCTTTCTTCCAC	IL
RM2-F	CAGTTGGCAATTGTCACGAG	
RM2-R	TCGAACATATCCAAGCCTCC	
